# The Urgency of Climate-Resilient Health Systems in Pakistan: Lessons From the 2022 Floods

**DOI:** 10.3389/ijph.2024.1607981

**Published:** 2024-11-01

**Authors:** Tayyab Mansoor Akthar, Michael J. A. Reid

**Affiliations:** ^1^ Global Health Department, Health Services Academy, Ministry of National Health Services, Regulations and Coordination, Islamabad, Pakistan; ^2^ Institute of Global Health Sciences, University of California at San Francisco, San Francisco, CA, United States

**Keywords:** Pakistan, climate, public health systems, floods, adaptation

The catastrophic floods that struck Pakistan in 2022 brought unprecedented devastation, revealing deep vulnerabilities in the country’s infrastructure and public health systems. As the waters receded, the full extent of the disaster became clear: 1700 people died, and one in seven Pakistanis, or around 33 million people, were directly impacted, including 8 million who were displaced from their homes [1]. Given the probability of future floods of similar severity [2], urgent action is necessary to ensure that Pakistan doesn’t experience the same disastrous consequences again. In this commentary, we review the impact of the 2022 floods and outline the importance of sustained investment in climate-responsive health systems in Pakistan and across East Asia.

Between June and October 2022, Pakistan experienced a sequence of unusually intense monsoon rainfall. The extreme rainfall caused widespread landslides along the Indus River basin, resulting in flooding across one-third of the country [3]. Beyond the immediate impact of flooding on lives and livelihoods, the floods also had a profound impact on access to healthcare services for the most vulnerable populations—children, the elderly, and those with chronic conditions. The floods destroyed 13% of the country’s medical facilities and left 15% of the population across all provinces without access to primary and secondary healthcare [1]. In addition, incident malaria increased fivefold to 2.6 million in the immediate aftermath of the floods [4]. Moreover, more than 41,000 dengue cases were reported later that year, mostly in Sindh and Balochistan, the disaster’s most severely affected districts [5]. Over 5.4 million people were dependent on contaminated water from ponds and wells as a result of the floods damaging the majority of the water systems in the areas that were affected. This led to a severe cholera outbreak, with almost 2,000 confirmed cases by the end of 2022 in all four provinces of Pakistan [6].

Economically, the impact was also profound. It is estimated that floods cost the Pakistan economy $15.2 billion in economic losses [7]. The agricultural sector, which forms the backbone of Pakistan’s economy, was devastated. Vast areas of farmland were submerged, leading to immediate food shortages and threatening long-term economic stability for farmers and rural communities. Malnutrition, which was already dangerously high in the country, was substantively increased. According to a survey by the UN’s Children’s Fund in 15 flood-affected districts released in May 2023 revealed that “one-third of children aged 6–23 months had suffered from moderate acute malnutrition, and 14% suffer from severe acute malnutrition, a life-threatening form of malnutrition [8].

Unfortunately, the initial public health response in the affected regions was undermined by logistic and administrative challenges, and health systems were quickly overwhelmed [9]. Rebuilding and reinforcing health infrastructure to be resilient against future climate shocks is imperative. Leveraging technology, like Sehat Kahani’s telemedicine platform, to provide remote healthcare access in disaster-affected areas [10] or using AI-powered predictive analytics and satellite data enhance flood risk assessment and early warning systems could enable rapid response to future climate-shocks. Lessons can also be learned from other climate-affected countries, such as Sri Lanka, which established a Climate Change and Health Unit within the Ministry of Health in 2016. This unit is vital in coordinating national efforts to address climate-related health risks, including conducting research, collecting data, and implementing climate adaptation strategies. A similar unit in Pakistan could serve as a centralized body to lead research initiatives, monitor climate-related health trends, and coordinate national adaptation strategies.

Given the limited domestic funding available for climate-related health adaptations and the burden of substantial sovereign debt, international donors and the private sector can play a critical role in helping governments build climate resilience. Facilitating access to climate financing mechanisms, including the World Bank’s Strategic Climate Fund, could enable Pakistan to undertake large-scale adaptation and mitigation projects that could protect against future climate-related disasters. The private sector also has a vital role to play in this effort. For example, Engro Corporation, one of Pakistan’s largest conglomerates, with diversified businesses spanning several key sectors, could promote renewable energy projects to reduce rural vulnerability to climate shocks; And K-Electric, Pakistan’s largest providers of electricity, could support the development of climate-resilient energy grids to ensure healthcare facilities remain operational during extreme weather events.

The catastrophic floods in Pakistan in 2022 underscore the urgent need for comprehensive, coordinated policy responses that addressed both immediate relief and long-term resilience. To avoid repeating the same mistakes again, it’s essential for government, donors, and international partners to work together to secure a safer, more sustainable future for Pakistan, turning this crisis into an opportunity to build back better and stronger. Bold and decisive action is needed to ensure that Pakistan could withstand future challenges and protect its people and economy from the devastating impacts of such disasters.
